# 

**Published:** 1871-12

**Authors:** 


Vol. XI.] DECEMBER, 1871. [No. 5.

### BUFFALO

### Medical and Surgical Journal.

EDITED BY JULIUS F. MINER, M. D.,

*Professor of Special Surgery in the Buffalo Medical College; Surgeon to the Buffalo General Hospital. etc., etc.*

BUFFALO.

PUBLISHED FOR THE EDITOR.

1871.

